# Implementing population-based mass drug administration for malaria: experience from a high transmission setting in North Eastern Uganda

**DOI:** 10.1186/s12936-019-2902-z

**Published:** 2019-08-09

**Authors:** Ronald Mulebeke, Humphrey Wanzira, Fred Bukenya, Thomas Eganyu, Kathryn Collborn, Richard Elliot, Jean-Pierre Van Geertruyden, Dorothy Echodu, Adoke Yeka

**Affiliations:** 1 Pilgrim Africa, Kampala, Uganda; 2 Makerere University School of Public Health, Kampala, Uganda; 3 Global Health Institute, University of Antwerp, Antwerp, Belgium; 4 University of Colorado Denver, Colorado, CO USA; 5 Boise State University, Boise, CO USA

**Keywords:** Malaria, Mass drug administration, High transmission setting, Uganda

## Abstract

**Background:**

Mass drug administration (MDA) is a suggested mean to accelerate efforts towards elimination and attainment of malaria-free status. There is limited evidence of suitable methods of implementing MDA programme to achieve a high coverage and compliance in low-income countries. The objective of this paper is to assess the impact of this MDA delivery strategy while using coverage measured as effective population in the community and population available.

**Methods:**

Population-based MDA was implemented as a part of a larger program in a high transmission setting in Uganda. Four rounds of interventions were implemented over a period of 2 years at an interval of 6 to 8 months. A housing and population census was conducted to establish the eligible population. A team of 19 personnel conducted MDA at established village meeting points as distribution sites at every village. The first dose of dihydroartemisinin–piperaquine (DHA-PQ) was administered via a fixed site distribution strategy by directly observed treatment on site, the remaining doses were taken at home and a door-to-door follow up strategy was implemented by community health workers to monitor adherence to the second and third doses.

**Results:**

Based on number of individuals who turned up at the distribution site, for each round of MDA, effective coverage was 80.1%, 81.2%, 80.0% and 80% for the 1st, 2nd, 3rd and 4th rounds respectively. However, coverage based on available population at the time of implementing MDA was 80.1%, 83.2%, 82.4% and 82.9% for rounds 1, 2, 3 and 4, respectively. Intense community mobilization using community structures and mass media facilitated community participation and adherence to MDA.

**Conclusion:**

A hybrid of fixed site distribution and door-to-door follow up strategy of MDA delivery achieved a high coverage and compliance and seemed feasible. This model can be considered in resource-limited settings.

## Background

Although the malaria burden is declining in several countries [1, 2], malaria-related morbidity and mortality remains high in sub-Saharan African countries, such as Uganda [1–6]. In the 2018 World Malaria Report, the sub-Saharan region contributed 92% of the global 219 million cases, with Uganda accounting for 4% of these cases. There are numerous efforts by the Uganda National Malaria Control Programme (UNMCP) to scale up recommended malaria control interventions including long-lasting insecticidal nets (LLINs), indoor residual spraying (IRS), intermittent preventive treatment in pregnancy (IPTp) and case management using artemisinin-based combination therapy (ACT). In spite of all the effort, malaria reports indicate a raising incidence in Uganda [7, 8].

Uganda, as a stably endemic, high burden country with pockets of extremely high malaria transmission faces the challenge of charting a rapid and safe route from a high malaria transmission zone towards pre-elimination phase via an intermediate low transmission state. In this regard there is a renewed interest in using malaria mass drug administration (MDA) to rapidly reduce the malaria burden and hasten the path to pre-elimination [9–11]. Whereas the WHO recommends use of MDA in low transmission settings [12], in the context of this study, MDA was used in combination with indoor residual spraying (IRS) to test how to accelerate reduction of malaria transmission. The impact of MDA on malaria burden will be reported elsewhere.

To impact on transmission, MDA requires high coverage of the target population which, in turn, demands a high level of community participation and engagement. MDA aims to provide therapeutic concentrations of anti-malarial drugs to a proportion of the population as large as possible in order to cure asymptomatic infections and to prevent re-infection during the period of post-treatment prophylaxis. MDA should be conducted in a coordinated manner, so that the drug is taken at approximately the same time by the whole population at risk, often at repeated intervals [13]. The current recommended MDA delivery strategies are door-to-door strategy and centralized, fixed site strategy [13]. Uganda has implemented MDA for river blindness, intestinal worms and onchocerciasis in communities and schools employing door-to-door strategy for the past 8 years [14]. In all these, MDA is delivered by village health team (VHT) and the drugs distributed are single doses not based on weight. MDA for malaria with a three-day weight-based regimen poses logistical challenges of ensuring appropriate dosing and completion. The choice of strategy may majorly depend on the local circumstances and should be defined bearing in mind the context for each area in country. The objective of this paper is to assess the impact of the MDA delivery strategy while using coverage measured as a proportion of the effective population and population available at the time of implementing MDA.

## Methods

MDA for malaria was administered in the framework of a clinical trial assessing the additional population impact of adding MDA to an IRS intervention in a high malaria transmission setting in Uganda (PACTR 201807166695568). Consent to participate was obtained from all participants prior to taking MDA. The study was approved by the Makerere University School of Public Health Higher Degrees Research Ethics Committee (MUSPH-HDREC) protocol reference number 327 and the Uganda National Council for Science and Technology (UNCST).

### Study setting

The study was conducted in Kapujan sub-county, Katakwi district, in North-Eastern Uganda. Kapujan sub-county lies along the shores of Lake Bisina, a fingerling of Lake Kyoga. The malaria prevalence in the area occurs all the year round. MDA was implemented in a study assessing the impact of population-based MDA given in combination with IRS or IRS alone. Four rounds of interventions were implemented over a period of 2 years. The first round was implemented in November–December 2016, the second in August 2017 the third from April–May 2018 and the fourth from November–December 2018.

### Study area and population

Farming is the main economic activity with occasional fishing. The area has 18 villages and each village has on average 5 to 6 village health team members (VHT). The VHTs serve to improve timely access to health care particularly for children under 5 years and pregnant mothers. The area has one health centre-III, and 2 heath centres IIs which are second and first line health units in the health care service delivery structure, respectively. The sub-county had universal mass distribution of LLINs in 2017 in addition to the study interventions.

### MDA implementation

#### Community engagement

Community awareness was created about MDA aimed at securing commitment and participation of all stakeholders. Community engagement commenced by meeting and sensitizing the district leadership including the District Health Officer, District Education Officer, the Chief Administrative Officer, the Resident District Commissioner, the District Malaria Focal Person and the District Health Management Team members. Community leaders were sensitized on the study objectives, interventions and the expected role of the community. Sensitization meetings were further held with sub county and village leaders. Community members were sensitized in village meetings. Information was provided about malaria prevention methods with a focus on MDA and its implementation. The rational of giving drugs to healthy people during MDA for prevention of malaria was emphasized. Community members were further informed on how to take the medicine, importance of completing the full dose, the expected drug reactions and the need to report these to the study team. During these meetings, study objectives, procedures, benefits and risks were comprehensively discussed and community members were given a chance to ask questions to which responses were made by the study team. Other mobilization strategies included radio announcements, radio spots, and use of mobile mega microphones. Interpersonal communication by the Local Council leaders (LCs) and VHTs continued throughout the implementation period.

#### Hiring and training study staff

A total of 108 VHTs, who are part of the existing health system structure, were recruited to help with community mobilization effort. In addition, 60 study staff were hired to work at distribution sites during implementation of MDA. Of these, 30 were data officers and 30 were health workers with variable training (nursing assistants, registered nurses, midwives, laboratory technicians and clinical officers). All were trained on performance of their roles at the distribution sites, conduct of the door-to-door follow up, and the study standard operating procedures (SOPs). All staff had a 4 days certified good clinical practice (GCP) training. The training included a pilot dry run. A team of 19 people conducted the exercise per village.

#### Enumeration of households

All households in the study area were mapped using hand held global positioning system (GPS) devices. Household members enumerated using hand held computers uploaded with Survey be software version (EDI-group, version 8, UK). Data was retrieved each day and stored on a central computer at the study office. Households and household members were assigned a unique identifier using a barcode system. Each individual’s barcode was attached to an individual card that is presented at the distribution site for individual verification. Individuals were screened to ascertain study eligibility. Children below 6 months and pregnant women were excluded as well as adults with history of chronic conditions like kidney and livers diseases. Consent to participate in the study was obtained from all potential study participants prior to implementation of MDA. Assent was obtained from children aged 8 to 17 years. Due to the dynamic nature of the study population, the enumeration database was updated before each round of the exercise. New households were mapped and members enumerated and screened. Old households were checked to update information in case some individuals left, died or new ones came in the study area and to update the eligibility of all household members.

#### Logistics and supplies

Team leaders ensured that all logistical supplies were available. A checklist was used to organize, issue and account for equipment and supplies designated for each fixed point. Each team was assigned a vehicle, 3 computers, 2 barcode scanners, 12 chairs and 6 tables, 3 Jerrycans, disposable cups and spoons, study drugs, stationery and laboratory supplies. The study drug was pre-packed according to weight-based doses and stored in a secure temperature controlled place.

#### MDA coordination

**Fig. 1:**
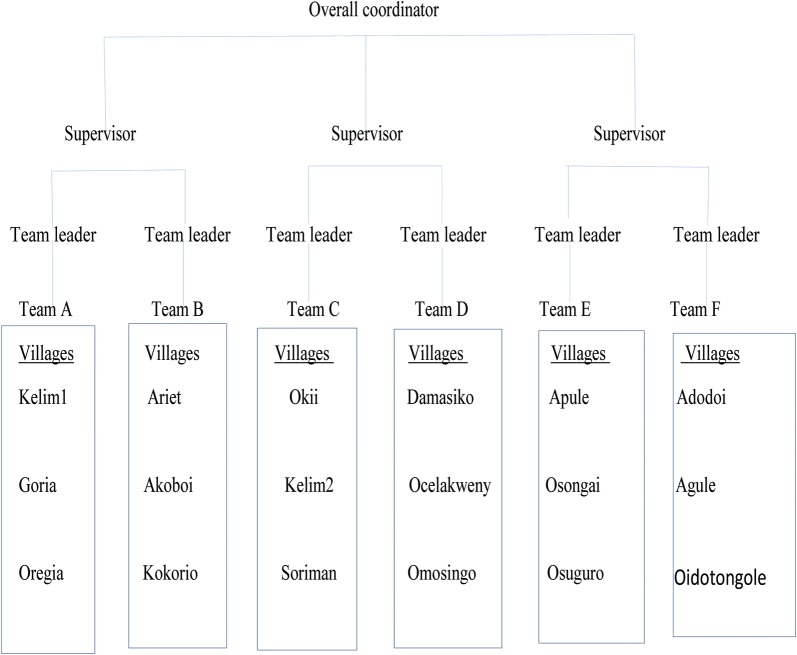
MDA implementation structure

The team comprised of an overall coordinator, sector supervisors, team leaders, health workers, data officers and VHTs (Fig. 1). A total of six teams were deployed to cover 18 villages over a period of 15 days. Each team had 19 personnel comprising a team leader, 5 health workers, 4 data officers and 9 VHTs. MDA was conducted by one team in each village over a period of 5 days. One sector supervisor was assigned to oversee 2 teams and ensured adequate supply of logistics and adherence to standard operating procedures (SOPs). Teams were systematically deployed in sequence. Each sector supervisor initiates one team at a time, after the second day of distribution, the second team would commence work in the next village. Once a team completes work over the 5 days, they would move to another village.

#### Fixed site distribution

**Fig. 2:**
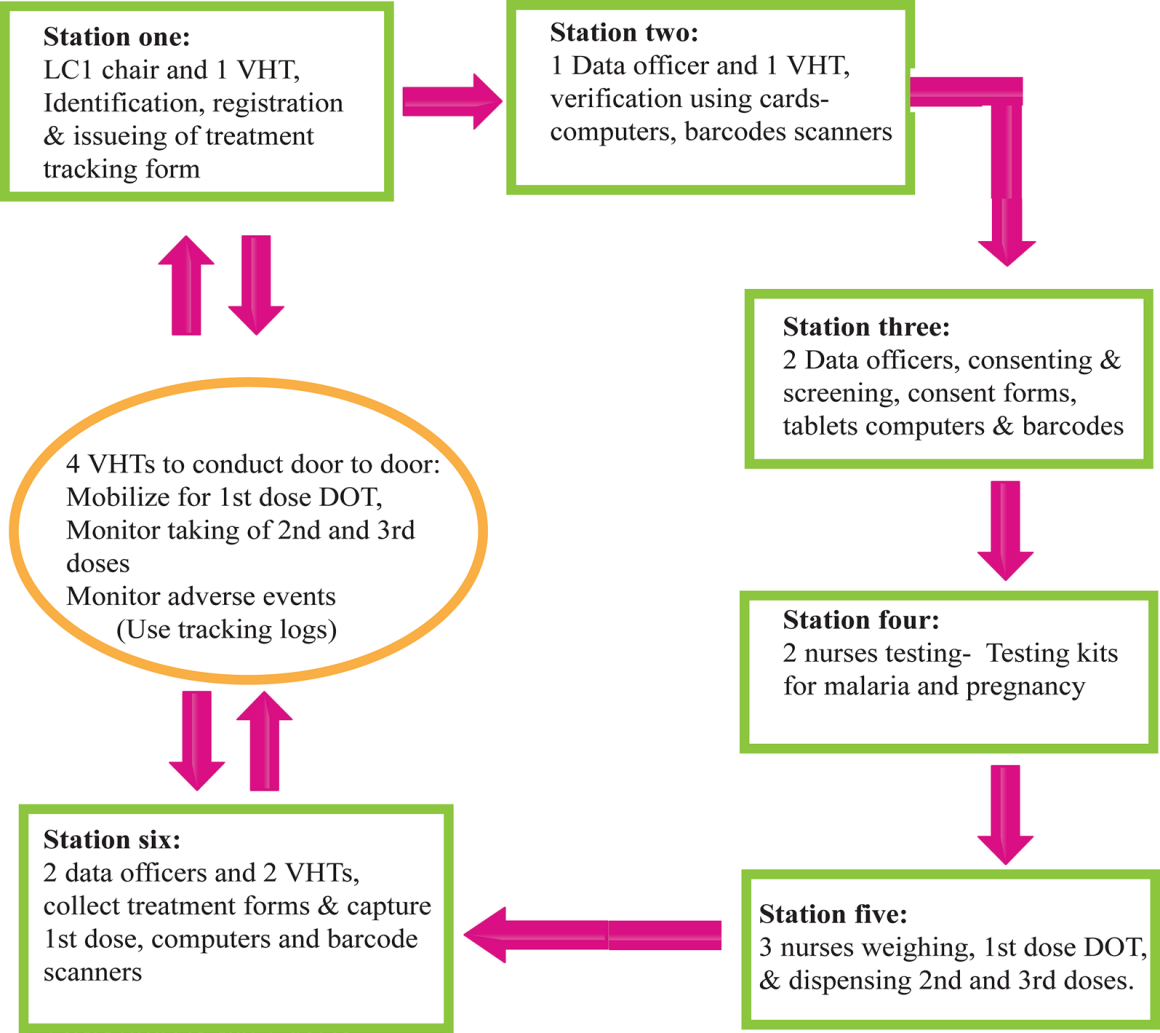
MDA distribution site map

**Fig. 3:**
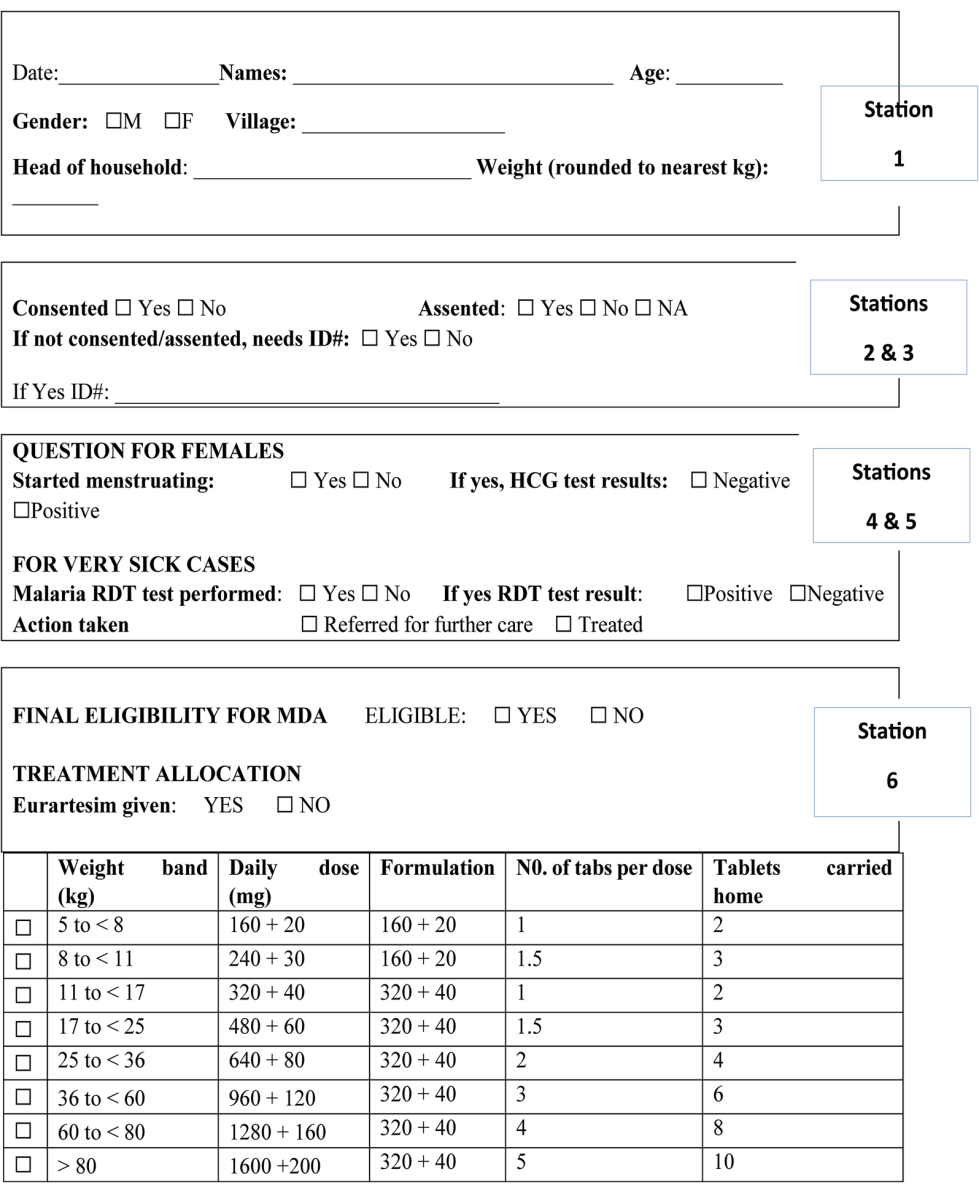
Malaria treatment tracking form

Fixed site delivery was implemented at established village meeting points in the study area for directly observed therapy (DOT) of the first dose. Village meeting points were known places for routine meetings in a village. At these meeting points, all eligible household members were mobilized to go for 1st DOT dose and to receive the 2nd and 3rd doses. Each static point had 6 stations numbered 1 to 6 and each had specific tasks (Figs. 2, 3).

Station one was the registration point where participants were registered. Following registration, MDA treatment tracking forms were issued to each participant and the participant’s particulars were written on the form.

Station two was participant’s identity verification point. Each participant presented their individual study identification card holding a unique barcode for verification. Two data officers, using barcode scanners and a computer verify each participant in the database, and confirm whether they were screened for eligibility criteria and had provided consent to participate in the study. On completion of the validating process, a mark was put on each participant’s card to indicate, the individual had reported to the distribution site. Those who had not consented during the enumeration exercise, were directed to station three to obtain informed consent, all information is recorded in the tracking form.

Station three was the point for seeking informed consent to participate in the study. Two study staff screened participants and sought for informed consent, for those who missed it during enumeration. The MDA treatment tracking form was updated accordingly.

At station four; two study nurses screened for pregnancy in every female participant of reproductive age (14–49 years). Urine samples were collected in urine containers and tested using HCG kits. Any participant who had a reactive pregnancy test was advised not to take the study drug and referred to attend antenatal care. The result was recorded on the MDA treatment tracking form. Participants whose HCG test was not reactive continued to station five to receive the study drug. Participants who had symptoms of malaria, were tested with a malaria RDT. The results (positive and negative) were recorded on the MDA treatment tracking form and all continued to station five.

At station five, three study nurses checked the eligibility of participants to take the study drug before dispensing the drugs. The study nurses checked for consent or assent to participate in MDA and results for urine HCG and malaria RDT tests where applicable. Those having a positive malaria RDT test received artemether–lumefantrine, the first-line treatment for uncomplicated malaria in Uganda, the rest received the study drug. Participant’s weight was taken and the study drug dispensed according to weight-based dosing. The first dose of treatment was given as DOT for each participant. The second and third doses were given to the participants with instructions to take the drugs at home.

At station six, data officers collected MDA treatment tracking forms from each participant. The barcode is scanned to update records of administration of the 1st DOT dose at site. This process updated the database instantly and provided information on real time coverage for 1st dose DOT. At the end of each day, a list of those who needed follow up to monitor adherence for the 2nd and 3rd doses was generated as well as those who still needed to be mobilized to come for the 1st dose.

MDA was conducted between 8th and 22rd December 2016 for round one, between 14th and 29th August 2017 for round two and 27th April to 10th May 2018 for round three and 27th November to 12th December for round 4.

#### Door-to-door follow up

On the second day of distribution in a village, about 4 VHTs started door-to-door monitoring for treatment adherence for the 2nd and 3rd dose and inquiring about any adverse events in each village. VHTs also mobilized individuals who did not turn up at the treatment points and encouraged them to report for treatment within the 4 days of distribution. They provided household members with telephone numbers to call without paying (hot lines), for communicating to the study team doctors in case of any symptoms after taking the study drug. Adherence was monitored by checking for empty drug blister packs. Tracking logs indicating participant’s completion of 2nd and 3rd doses were filled for each participant in a village. Participants were followed up in each village for extra 2 days to record the taking the 2nd and 3rd doses.

#### Management of adverse events

Expected drug reactions and side effects were explained to the community members at village meetings and by use of radio talk shows. This communication was continuously provided during and after MDA implementation. Two hotlines for calling study doctors were circulated to community members during the meetings. When a community member felt unwell, they contacted the medical team through the hot lines, the team assessed whether the participant experienced an adverse event or a drug reaction. The medical team moves to the households to assess such study participants. Depending on the assessment of the doctors, a vehicle was availed to transport patients who needed to be managed in a health facility. Both health facility staff and the study team doctors worked together to manage adverse events All complaints were documented using a standard adverse event reporting form from the study drug manufacturer (Sigma Tau) and the institutional review board (IRB). All adverse events were managed following the standard of care and were followed until they resolved. Prior to implementing interventions, health facility staffs were trained in management of possible adverse events and complications of the study drugs that were likely to be reported. Drugs and other supplies were fully stocked to ensure appropriate case management according to the country’s treatment guidelines.

## Results

### Enumeration of study participants

The area has a population of 14,468 people based on study enumeration data. About 52.0% of the population is females and 57.0% are children under 15 years. The area has 2490 households with average house occupancy of 5.5 persons per household.

### MDA coverage

MDA coverage was estimated based on enumeration updates, which were conducted before commencement of interventions. Success of this delivery strategy was measured by using effective coverage (coverage based on all individuals who lived in the in past 6 months and plan to live in the same community in the next 6 months.) and coverage based on population available during the implementation of MDA. For effective coverage, MDA coverage was 80.1%, 81.2%, 80.0% and 80% for first, second, third and fourth rounds of MDA respectively (Table 1). Whereas coverage based on population available was 80.1%, 83.2%, 82.4% and 82.9% % for first, second, third and fourth rounds, respectively. Coverage for each round was based on number of individuals who turned up at the distribution sites against number of individuals enumerated in the study area. Coverage at village level and factors affecting uptake of MDA in this setting is described elsewhere. Adherence for round one was not monitored as the implementation had an adaptive approach to try and find a strategy that would adequately yield satisfactory results. For instance, the process was improved by introducing monitoring of uptake of 2nd and 3rd doses using tracking logs. Consequently, adherence to subsequent rounds was over 80% for second and third doses. Lessons learn from round one were used to improve subsequent rounds.

**Table 1:** Mass drug administration coverage in Kapujan sub-county, Katakwi district

	Round 1 2016		Round 2 2017		Round 3 2018		Round 4 2018	
	N = 15,639^a^		N = 15,543^a^		N = 15,450^a^		N = 15,559^a^	
Fixed distribution (DOT): number treated and percentage, 95% CI								
Dose 1, DOT	12,523^b^	80.1 (0.794–0.807)	12,620^b^	81.2 (0.806–0.818)	12,366^b^	80.0 (0.794–0.807)	12,4449^b^	80.0 (0.794–0.806)
Door-to-door monitoring: number treated and percentage								
Dose 2	–	–	12,488	80.1 (0.795–0.807)	12,344	79.9 (0.793–0.805)	12,399	79.7 (0.791–0.803)
Dose 3	–	–	12,444	80.1 (0.794–0.807)	12,343	79.9 (0.793–0.805)	12,399	79.7 (0.791–0.803)
Overall		80.1 (0.794–0.807)		81.2 (0.806–0.818)		80.0 (0.794–0.807)		80.0 (0.794–0.806)

^a^Number of individuals living in the study area at the time of interventions

^b^Number of individuals eligible to receive the study drug

### Lessons learnt

Engaging the leadership at all levels provided community acceptance and improved willingness to participate in MDA. Using VHTs, and local council leaders, in community mobilization, built confidence in the community about MDA. Interpersonal communication by door-to-door mobilization conducted by local council leaders and VHTs was very effective in mobilizing the community to participate in the exercise.

Hiring local staff who knew the local language, facilitated effective communication between study staff and study participants. Training study staff equipped them with the necessary knowledge and acceptable practices for handling study participants. Working with the community promoted ownership as everyone desired to achieve a high coverage in their area.

Preparing logistics in time before commencement of field activities was very important. Teams assigned to villages ensured high turn up at distribution sites and satisfactory follow up of 2nd and 3rd doses. This approach did not only improve monitoring MDA activities but provided an opportunity for team work within each team. Team work resulted in commitment by the study team, local leadership and VHTs to ensure coverage is high in villages which were assigned to them. It provided for adequate accountability for study drugs as well as logistical supplies.

Fixed site distribution and door-to-door follow up was effective in achieving a high MDA population coverage. It facilitated close supervision to ensure adherence to SOPs and participant safety. The delivery of drugs, test kits, weighing scales, computers, scanners, treatment forms and other supplies to the distribution sites was logistically feasible compared to when study staff would move door-to-door with all these items. It is a logistically feasible approach for delivering MDA because of employing fewer personnel and minimal transport needs. This approach both accommodated our resource constraints and served as a knowledge sharing and capacity building process within the research team.

### Challenges

Additionally, implementing MDA during the rainy season is challenging because most community members were busy in their gardens. Inadequate duration of engagement with the community before and during implementation of MDA. This approach needed a large amount of investment put in mobilizing the community.

## Discussion

Population-based MDA was implemented using dihydroartemisinin-piperaquine (DHA-PQ) delivered by fixed point distribution for the first dose and door-to-door follow up for the second and third doses in Kapujan sub-county, an area of high malaria transmission in North Eastern Uganda. Of the eligible persons at each round of intervention, MDA coverage was 80.1%, 81.2%, 80.0% and 80% for the 1st, 2nd 3rd and 4th rounds, respectively. Treatment adherence to all three doses in round one, was not captured but for subsequent rounds was over 80% for second and third doses. Intense community mobilization using community structures and mass media facilitated community participation and adherence to MDA.

Community engagement was very critical during the implementation process of MDA. Engaging the community about the importance of the project prior to implementation of MDA increased participation among the local leadership and the community members. Findings are similar to that in the Gambia, where sensitizing the community increased participation in MDA project [15]. Use of a collaborative approach to mobilize the community by the study team in partnership with the local leadership ensured community participation and commitment to MDA implementation. This similarly reported in Cambodia, [16] where community health workers, community leaders and political leaders took a central role in community mobilization for a successful MDA implementation. Active community engagement, as recommended by the WHO, is essential for any MDA implementation plan.

Updating the household and population database before each round of MDA provided an accurate population size to target for each round of MDA and enabled a more accurate estimate of MDA coverage in the community. A literature review of other studies reports unclear and inconsistent methods for estimating coverage [17]. Furthermore, screening for MDA eligibility and getting participant consent during the enumeration process improved efficiency of the exercise. This exercise was closely monitored by the study internal systems to ensure that the information obtained about participants was accurate and that there was no coercion to participate.

To carry out an organized MDA across all distribution sites, a structure was developed to facilitate quick access to logistics, close supervision and accountability of study supplies. With little documentation of similar structures in other MDA settings, this structure may provide a working framework for organizing MDA distribution sites in comparable settings in low-income countries.

A framework was created to allow consistent MDA implementation at the distribution sites from station 1 to 6. This allowed MDA implementation in a consistent manner across all distribution sites. In the literature reviewed, it was not possible to find any study with such a flow plan, but the algorithm created was significant in ensuring consistency in methodology and standardization of activities at distribution sites. Door-to-door follow up by VHTs was key in ensuring adherence to the 2nd and 3rd doses. The door-to-door follow up process provided continuous contact between the individual participants and study team post 1st dose DOT. The collection of blister packs after each individual has taken their 2nd and 3rd doses was in some way to validate the number of doses given out and those taken.

Fixed site distribution for 1st dose and door-to-door monitoring of 2nd and 3rd doses was successful in achieving a simultaneous high population coverage within a period of 15 days. The proportion of the effective population that took the weight-specific dose at distribution sites under direct observation therapy (DOT) was 80.1%, 81.2%, 80% and 80% for rounds 1, 2, 3 and 4 respectively. About 80.1%, 79.9% and 79.7% % reported taking 2nd and 3rd doses during the door-to-door follow up for MDA rounds 2, 3 and 4, respectively. The strategy of door-to-door follow up could be among other factors contributing to ensuring participants complete the 3-day’s regimen compared to a fixed point distribution alone. MDA implemented in two zones in Liberia during the Ebola outbreak documented much lower coverage of 52% and 22% for round 1 and 2, respectively [18]. In a study in Eastern Myanmar, fixed site distribution using malaria posts, MDA participation achieved was slightly over 60% in smaller villages and registered below 30% in larger villages [19]. In comparing to door-to-door delivery with age-specific doses conducted in a study in Sierra Leone [20], coverage of DOTs was 71% and 97.1% coverage in a Zanzibar [21], while in the hybrid model in this study DOTs was 81% and 76% and 80% in rounds 1, 2, and 3, respectively. Outwardly, a combination of fixed site distribution with door-to-door follow up when administering weight-specific doses, promotes compliance and is logistically feasible for reaching a large population at the same time. The WHO puts more emphasis on the door-to-door MDA delivery strategy [13], which may work well with age-specific doses although the logistics for its roll out are not well documented. It is likely that in population-based MDA, logistics of carrying computers, weighing scales, testing kits for pregnancy and drugs may be an issue if door-to-door MDA delivery is to be implemented. A combination of fixed distribution and door-to-door follow up strategy is recommended for population-based MDA especially in countries with a similar setting.

As much as guidelines or recommendations mention about pharmacovigilance in MDA, how to practically implement it is not well documented. In this study, a field medical team in collaboration with health workers from health centres in the study area managed the adverse events (AE). A study by Landier et al. reported use of a medical team with a mobile clinic for monitoring and management of adverse events [22] during MDA. In this study, hot lines for reporting adverse events to the medical team were used. Management of adverse events was carried out at the health facilities where drugs were stocked, health workers were trained on management of adverse events and proper documentation of adverse events was done. This provided a logistically feasible and an efficient surveillance system for monitoring adverse events as existing structures in the health system were used.

Timing for MDA implementation should target school holidays and before the rainy season starts when the community is less mobile [23]. Round one and round four of MDA implementation was during the December dry season and when school children were back home for holidays, a high coverage was attained during these rounds. A similar observation was made in The Gambia [15] and they recommend that MDA activities be undertaken just before the rainy season.

The logistical complexities for implementing MDA may need to change from place to place and from time to time. The tools for MDA implementation need to allow for sufficient flexibility to be adapted by all users in varying settings. With the dynamics of the population, there will always be new challenges and new ways of coping.

## Limitations

Although, MDA implementation was a success, several limitations exist. In 2 out of the 3 rounds of implementation, MDA activities were conducted during the school term, making it difficult to reach school children. At the same time, the community was mobile due to activities associated with the rainy season. LLIN and IRS campaign conducted in the area could have influenced compliance to MDA as the community could feel protected. This could have also introduced intervention fatigue in the community. Lack of capacity to closely monitor migration of the population in and out of the study area may have resulted into underestimation or overestimation of MDA coverage. Managing perceptions of drug side effects was complex as it was difficult to get everyone understand the difference between side effects and adverse events. It was difficult to explain malaria transmission in relation to the need to take drugs for a person who is not sick.

## Conclusion

Using community structures for community sensitization and mobilization facilitates high participation in MDA. Updates of mapping and enumeration database before each round of interventions is essential for accurate estimate of coverage. Screening and consenting during each round is key for identification of eligible participants. Organizing teams based on number of villages and available resources helps to manage logistical issues and supervision of processes. Fixed site distribution design, built on existing resources allows MDA site distribution to be implemented in a consistent and standard manner across all distribution sites for 1st dose under DOT. Door-to-door follow up by VHTs is key in ensuring adherence to 2nd and 3rd doses and timely identification and management of adverse events. A hybrid (Box 1) of fixed site distribution for 1st dose under DOT and door-to-door monitoring promotes and simultaneously allows assessment of adherence to and safety of the 3 days ACT regimen.

**Table:** Box 1: Summary of MDA distribution activities

Fixed site distribution Organizing the site prior to distribution Verifying eligibility to take MDA Screening of women of reproductive age for pregnancy Registration of individuals receiving the study drug Distribution of blisters with 1st dose under DOT Tally sheet completed after medicine has been dispensed Monitoring for serious adverse events or Adverse events for at least 30 min and Referral of all ill people to the nearest health facility	Door-to-door distribution Verification of household members Explaining of objectives for the campaign Obtained consent/assent Checking for eligibility Screening for pregnancy among women of reproductive age Distribution of blisters appropriate for the age category Give instruction for taking the remaining Doses on days two and three Mark the tally sheet Mark the completed house hold
Mix of two approaches Organizing the sites to have facilities like, pit latrines, tables, chairs, drinking water, shelter or waiting shades Identification and verification of village members Consenting and/or assenting Screening for eligibility Testing for pregnancy Dispensing of drugs All ill people referred to health facilities Door-to-door follow up using tracking logs to identify people who should be mobilized To go to the site for day 1 dose under DOT Monitored the taking of 2nd or 3rd doses Inquired about adverse events	

## Data Availability

Dataset used for this manuscript are submitted.

## Abbreviations

ACT: artemisinin-based combination therapy
AE: adverse event
BCC: Behaviour Change and Communication
DOT: direct observation therapy
GCP: good clinical practice
IEC: information education & communication
IPTp: intermittent preventive treatment in pregnancy
IRS: indoor residual spraying
LC1: local council 1
LLIN: long-lasting insecticide treated net
MDA: mass drug administration
MOH: Ministry of Health
RDT: rapid diagnostic test
SAE: serious adverse event
SOP: standard operating procedure
SPH-HDREC: School of Public Health Higher Degrees Research & Ethics Committee
UNCST: Uganda National Council of Science & Technology
UNMCP: Uganda National Malaria Control Programme
VHT: village health team
WHO: World Health Organization

## References

[1] Mukonka VM, Chanda E, Haque U, Kamuliwo M, Mushinge G, Chileshe J (2014). High burden of malaria following scale-up of control interventions in Nchelenge District, Luapula Province, Zambia. Malar J..

[2] Assele V, Ndoh GE, Nkoghe D, Fandeur T (2015). No evidence of decline in malaria burden from 2006 to 2013 in a rural Province of Gabon: implications for public health policy. BMC Public Health..

[3] Ashley EA, Pyae Phyo A, Woodrow CJ (2018). Malaria. Lancet.

[4] Okiro EA, Kazembe LN, Kabaria CW, Ligomeka J, Noor AM, Ali D (2013). Childhood malaria admission rates to four hospitals in Malawi between 2000 and 2010. PLoS One.

[5] Jagannathan P, Muhindo MK, Kakuru A, Arinaitwe E, Greenhouse B, Tappero J (2012). Increasing incidence of malaria in children despite insecticide-treated bed nets and prompt anti-malarial therapy in Tororo, Uganda. Malar J..

[6] Okiro EA, Bitira D, Mbabazi G, Mpimbaza A, Alegana VA, Talisuna AO (2011). Increasing malaria hospital admissions in Uganda between 1999 and 2009. BMC Med..

[7] WHO (2017). World malaria report 2017.

[8] WHO (2018). World malaria report.

[9] Gosling RD, Okell L, Mosha J, Chandramohan D (2011). The role of antimalarial treatment in the elimination of malaria. Clin Microbiol Infect.

[10] Achan J, Mwesigwa J, Edwin CP, D’Alessandro U (2018). Malaria medicines to address drug resistance and support malaria elimination efforts. Expert Rev Clin Pharmacol..

[11] Nosten F (2016). Elimination in South-East Asia? The role of antimalarial drugs (in French). Bull Acad Natl Med..

[12] Bosman A. Mass drug administration: WHO policy update. Bogota: World Health Organization, World Malaria Programme, 3–5 May 2016.

[13] WHO. Mass drug administration for falciparum malaria: a practical field manual. Geneva: World Health Organization; 2017.

[14] Katabarwa MN, Griswold E, Habomugisha P, Eyamba A, Byamukama E, Nwane P (2019). Comparison of reported and survey-based coverage in onchocerciasis programs over a period of 8 years in Cameroon and Uganda. Am J Trop Med Hyg.

[15] Dial NJ, Ceesay SJ, Gosling RD, D’Alessandro U, Baltzell KA (2014). A qualitative study to assess community barriers to malaria mass drug administration trials in The Gambia. Malar J..

[16] Peto TJ, Tripura R, Davoeung C, Nguon C, Nou S, Heng C (2018). Reflections on a community engagement strategy for mass antimalarial drug administration in Cambodia. Am J Trop Med Hyg.

[17] Adhikari B, James N, Newby G, von Seidlein L, White NJ, Day NP (2016). Community engagement and population coverage in mass anti-malarial administrations: a systematic literature review. Malar J..

[18] Kuehne A, Tiffany A, Lasry E, Janssens M, Besse C, Okonta C (2016). Impact and lessons learned from mass drug administrations of malaria chemoprevention during the Ebola outbreak in Monrovia, Liberia, 2014. PLoS One.

[19] Landier J, Kajeechiwa L, Thwin MM, Parker DM, Chaumeau V, Wiladphaingern J (2017). Safety and effectiveness of mass drug administration to accelerate elimination of artemisinin-resistant falciparum malaria: a pilot trial in four villages of Eastern Myanmar. Wellcome Open Res..

[20] Aregawi M, Smith SJ, Sillah-Kanu M, Seppeh J, Kamara AR, Williams RO (2016). Impact of the Mass Drug Administration for malaria in response to the Ebola outbreak in Sierra Leone. Malar J..

[21] Ali AS, Thawer NG, Khatib B, Amier HH, Shija J, Msellem M (2017). Artemisinin combination therapy mass drug administration in a setting of low malaria endemicity: programmatic coverage and adherence during an observational study in Zanzibar. Malar J..

[22] Landier J, Parker DM, Thu AM, Lwin KM, Delmas G, Nosten FH (2018). Effect of generalised access to early diagnosis and treatment and targeted mass drug administration on *Plasmodium falciparum* malaria in Eastern Myanmar: an observational study of a regional elimination programme. Lancet.

[23] Gerardin J, Bertozzi-Villa A, Eckhoff PA, Wenger EA (2018). Impact of mass drug administration campaigns depends on interaction with seasonal human movement. Int Health..

